# Ultra-high sensitive 1D porous silicon photonic crystal sensor based on the coupling of Tamm/Fano resonances in the mid-infrared region

**DOI:** 10.1038/s41598-019-43440-y

**Published:** 2019-05-06

**Authors:** Ashour M. Ahmed, Ahmed Mehaney

**Affiliations:** Physics Department, Faculty of Science, Beni-Suef University, Beni-Suef, 62514 Egypt

**Keywords:** Biophotonics, Photonic devices

## Abstract

Porous silicon one-dimensional photonic crystals (PSi-1DPCs) are capable of sensing solutions and liquids based on the smallest variation of the refractive indices. In the present work, we present a novel metal/PSi-1DPC as a liquid sensor based on Tamm/Fano resonances. The operating wavelength range is from 6.35 to 9.85 μm in the mid-infrared (MIR) spectral region. Different metals (Al, Ag, Au, and Pt) are attached to the top surface of the PSi-1DPCs structure to show Tamm/Fano resonances more clearly. To the best of our knowledge, it is the first time that Tamm/Fano resonances exhibit simultaneously in PSi-1DPCs within the same structure. The reflection spectra were calculated for the metal/PSi-1DPC structure by using the transfer matrix method (TMM) and the Bruggeman’s effective medium approximation (BEMA). The simulations show that the Tamm/Fano resonances are red-shifted towards the higher wavelengths with increasing the refractive index of the pores. The Ag/PSi-1DPC sensor showed the highest performance. Its sensitivity can be reached to the value 5018 nm/RIU with a high-quality factor of about 2149.27. We predict the proposed sensors can be easily fabricated and we expect them to show higher performance than other reported sensors of this type. Therefore, it will be of interest in the field of optical sensing in different fields.

## Introduction

Photonic crystals (PCs) are new smart composite structures could control the propagation of electromagnetic waves with different frequencies. The novel phenomenon appeared on PCs is the photonic band gap^1–7^. Within the band gap, all frequencies of the incident electromagnetic waves are effectively attenuated. Breaking the periodicity of PCs and generating resonant modes inside the photonic band gaps increase the novelty of such periodic structures more than the perfect ones. Based on these remarkable physical and engineering applications, PCs inspired researchers to replicate this idea on different types of waves e.g., elastic, acoustic, surface, electric and magnetic waves^8–13^. Recently, the process of solutions and liquids sensing by using PCs has attracted great attention due to its major role in many biological, biochemical and engineering applications^14–21^.

On the other hand, the porous silicon (PSi) can be used as a biosensor due to it has a considerable surface area and versatile surface chemistry^22,23^. The PSi is a form of the silicon wafer that has introduced nanopores in its microstructure. It can be easily formed by electrochemical etching of silicon wafers in hydrofluoric acid (HF) electrolyte under an applied electric current^24,25^. The electrochemical process allows for precise control over the properties of PSi such as porosity and thickness of the porous layer by adjusting the etching current density, time interval, and HF concentration^26^.

The one- dimensional photonic crystal (1DPC) based on multilayered PSi can be easily fabricated by periodic alteration of the current density during the electrochemical process^27^. The PSi-1DPC offers many advantages such as its high specific surface area, its ease of fabrication and compatibility with standard microelectronics processing^27–29^. R. Caroselli *et al*. experimentally developed a highly sensitive PC sensor based on PSi for low refractive index variations^30^. The PSi sensor showed a sensitivity of about 1000 nm/RIU. Also, V. Pham *et al*. developed a 1DPC microcavity sensor based on PSi multilayers^31^. The 1DPC sensor showed a sensitivity of about 200 nm/RIU, and it can be used for the determination of organic content in different liquid solutions.

In addition to that, the well-known phenomenon Tamm resonance has been achieved in PCs and it has been used in the optical sensing techniques of PCs structures^32–34^. Mainly, Tamm resonance is formed at the metal and the Bragg mirror interface. B. Auguie *et al*. studied theoretically and experimentally Tamm resonance in a PC composed of SiO_2_ and TiO_2_ multilayers^35^. The sensitivity of this structure was low of about 55 mm/RIU.

Moreover, Fano resonance was observed in many 1DPC structures, and it can be used as an indicator for many applications such as bio-sensing, switching, photodetector, filter, waveguide, and modulator^36–39^. V. Klimov *et al*. proposed theoretically Fano resonances in a 1DPC structure composed of MgF_2_ and TiO_2_ layers, the structure was used as a biosensor^40^. The proposed structure has achieved a wavelength sensitivity of about 17 nm/RIU.

In this work, we present a new theoretical study concerning Tamm/Fano resonance in a PSi-1DPC structure. The PSi-1DPC is a ternary structure composed of three different PSi layers repeated in N = 25 unit cells with a metal layer on the top of the ternary structure; Si/[PSi_1_/PSi_2_/PSi_3_]^N=25^/metal. Also, we will study the optical properties and sensitivity of the proposed metal/PSi-1DPC sensor. The paper is organized as follows; In the first, we present the design of a metal/PSi-1DPC sensor. Secondly, the theoretical basis equations of PSi and its relation with the refractive index of the void (the material filling the pores) are presented. Finally, the output simulated results and the sensitivity analysis of the metal/PSi-1DPC sensor are introduced and discussed.

## Results

### Design of PSi-1DPC sensor

**Figure 1:**
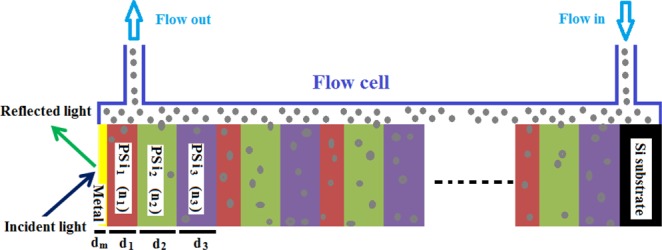
A schematic diagram of the metal/PSi-1DPC sensor structure.

The proposed configuration of the biosensor is a ternary Si/[PSi_1_/PSi_2_/PSi_3_]^N=25^/metal as shown in Fig. 1. Recently, the periodic ternary 1D-1DPC structures have attracted the researchers attention due to their high sensing performance compared with the binary systems^41,42^. For refractive index detection, the target solution is injected inside the flow cell in contact with the top surface of the PSi-1DPC in order to allow for the solution to fill the pores as shown in the schematic diagram of Fig. 1. For an optimization purpose, the number of periods is chosen to be N = 25. Rea has fabricated experimentally and determined the porosity ratio in the PSi layer in the range from 56% to 81%^25^. Therefore, the calculated porosity here in this paper was determined and confirmed experimentally for the proposed PSi layers. The values of the different parameters of the materials used in the present simulation are given in Table 1. These values are given after optimization to get the best sensor performance. See Table S1 in the supplementary data.

**Table 1:** The values of the different parameters of the materials used in the calculations.

Layer	Porosity (%)	Thickness (µm)
First layer (PSi_1_, n_1_)	65	d_1_ = 0.5
Second layer (PSi_2_, n_2_)	77	d_2_ = 1.0
Third layer (PSi_3_, n_3_)	85	d_3_ = 1.0
Metal	Ag, Al, Au, and Pt	d_m_ = 0.040

The transfer matrix method (TMM) is a suitable method for calculating the reflection of the multilayer structure^1–5^. From the TMM we obtain the reflectance of electromagnetic waves through metal/PSi-1DPC as follows^43^:

1 $${\rm{R}}=100{|\frac{({{\rm{M}}}_{11}+{{\rm{M}}}_{12}{{\rm{q}}}_{{\rm{s}}})\ast {{\rm{q}}}_{0}-({{\rm{M}}}_{21}+{{\rm{M}}}_{22}{{\rm{q}}}_{{\rm{s}}})}{({{\rm{M}}}_{11}+{{\rm{M}}}_{12}{{\rm{q}}}_{{\rm{s}}})\ast {{\rm{q}}}_{0}+({{\rm{M}}}_{21}+{{\rm{M}}}_{22}{{\rm{q}}}_{{\rm{s}}})}|}^{2}$$

where M_11_, M_22_, M_12_ and M_21_ are the elements of the total transfer matrix of the proposed structure. The q values of air and substrate are given by the equations q_0_ = n_0_ cosθ_0_ and q_s_ = n_s_ cosθ_s_, respectively.

The angles of incidence of each layer are related to the angle of incidence θ_0_ in the air by Snell’s law as follows,

2 $${{\rm{n}}}_{0}\,{\sin {\rm{\theta }}}_{0}={{\rm{n}}}_{{\rm{i}}}\,{\sin {\rm{\theta }}}_{{\rm{i}}},\,{\rm{i}}=1,2,3,{\rm{m}},{\rm{s}},$$

where the order numbers 1, 2, 3, 4 and 5 represent the PSi_1_, PSi_2_, PSi_3_, metal, and substrate, respectively.

The detailed analysis of the TMM and the characteristics matrix details can be found extensively in literatures^4,36^.

### Porosity/refractive index relations of PSi

The effective refractive index of the PSi layer can be obtained by the Bruggeman’s effective-medium approximation (BEMA) as follows^27,44^:

3 $${\rm{P}}\frac{{{\rm{n}}}_{{\rm{v}}}^{2}-{{\rm{n}}}_{{\rm{eff}}}^{2}}{{{\rm{n}}}_{{\rm{v}}}^{2}+2{{\rm{n}}}_{{\rm{eff}}}^{2}}+(1-{\rm{P}})\frac{{{\rm{n}}}_{{\rm{Si}}}^{2}-{{\rm{n}}}_{{\rm{eff}}}^{2}}{{{\rm{n}}}_{{\rm{Si}}}^{2}+2{{\rm{n}}}_{{\rm{eff}}}^{2}}=0,$$

where n_Si_, n_v_ and n_eff_ are the refractive indices of silicon, the material inside the pore and the whole PSi layer, respectively. P is the porosity ratio.

Equation 3 can be rewritten in a new form to shows n_eff_ as a function of P and n_v_ as follows:

4 $${{\rm{n}}}_{{\rm{eff}}}=0.5\sqrt{3{\rm{P}}({{\rm{n}}}_{{\rm{v}}}^{2}-{{\rm{n}}}_{{\rm{Si}}}^{2})+(2{{\rm{n}}}_{{\rm{Si}}}^{2}-{{\rm{n}}}_{{\rm{v}}}^{2})+\sqrt{{(3{\rm{P}}({{\rm{n}}}_{{\rm{v}}}^{2}-{{\rm{n}}}_{{\rm{Si}}}^{2})+({{\rm{n}}}_{{\rm{Si}}}^{2}-{{\rm{n}}}_{{\rm{v}}}^{2}))}^{2}+8{{\rm{n}}}_{{\rm{Si}}}^{2}{{\rm{n}}}_{{\rm{v}}}^{2}.}}$$

For porosities 65%, 77%, and 85%, the corresponding refractive indices at a wavelength 8 μm are 1.6826, 1.381 and 1.2199, respectively.

**Figure 2:**
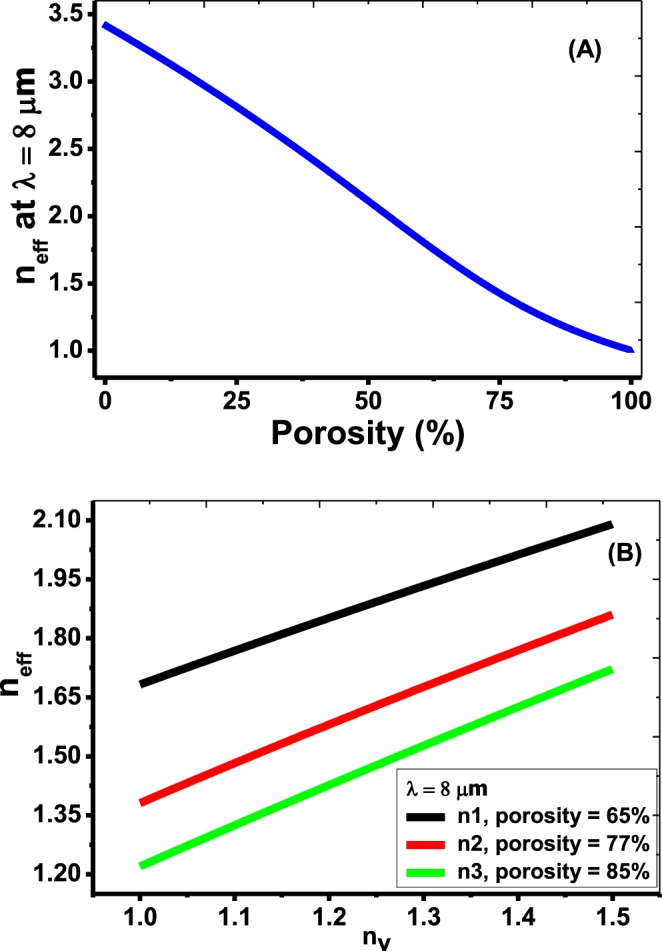
Change of the effective refractive index of the PSi layer as a function of (**A**) porosity ratio, (**B**) void refractive index n_v_.

According to Bruggeman’s model, Fig. 2(A) shows the dependence of the effective refractive index of the PSi on the porosity variation. With increasing the porosity value, the effective refractive index of the PSi will be decreased for all wavelength values in the range from 6.35 to 9.85 μm. Therefore, the effective refractive index of the PSi can be controlled from the range 3.4203 to 1 based on the porosity variation. Also, According to Fig. 2(B), n_eff_ increases linearly with increasing the refractive index of voids for the three porosities. Also, it is well-known that the effective refractive index does not vary in the mentioned operating wavelength range.

## Discussion

### Perfect PSi-1DPC

The reflectance of the incident electromagnetic wave for the perfect PSi-1DPC; Si/[PSi_1_/PSi_2_/Psi_3_]^N=25^ is plotted versus the incident wavelength. A ray of light is normally incident on the PSi-PCs structure (with an angle of incidence = zero degrees). As shown in Fig. S2 in the supplementary data, the reflectance was calculated at different void refractive indices (n_v_ = 1 to 1.5). As shown in this Figure, wide photonic band gaps appeared for each n_v_ value. These gaps resulted from the constructive interference of electromagnetic waves at the interface between each two layers^1,5,12^, since the width of the band gap increases with increasing the mismatch between the constituent materials^10–12^. With increasing the refractive index of the void, the photonic band gap decreases due to decrement the contrast of the refractive indices between the PSi and Si substance. Also, the spectrum is red-shifted towards the higher frequency range with increasing n_v_ value.

### Metal/PSi-1DPC sensor

**Figure 3:**
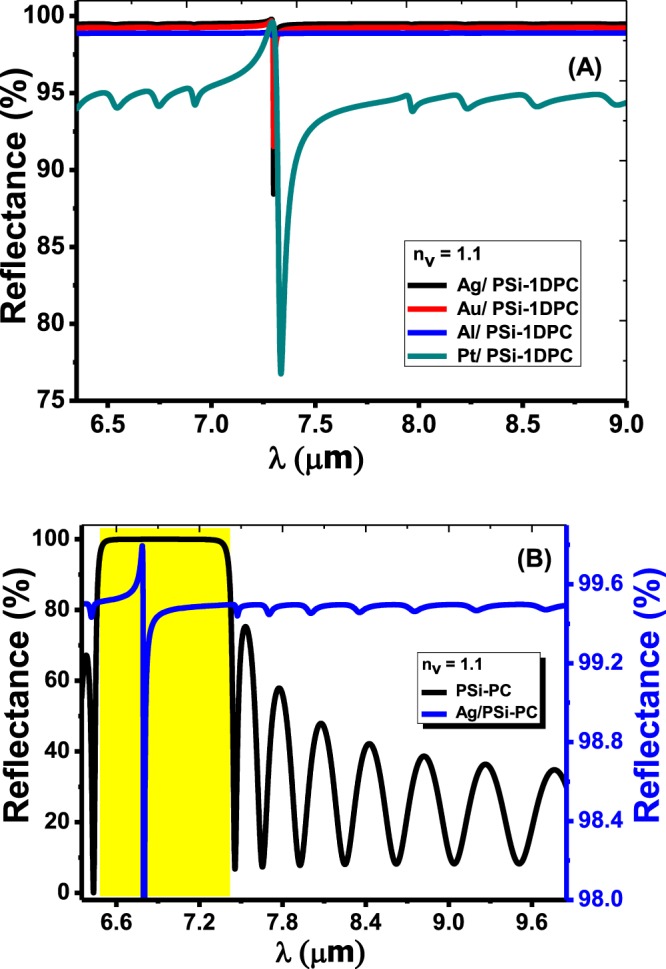
(**A**) The reflectance of the Ag/PSi-1DPC, Au/PSi-1DPC, Al/PSi-1DPC and Pt/PSi-1DPC structures as a function of the wavelength at n_v_ = 1.1, respectively. (**B**) A comparison between the reflectance of the PSi-1DPC and Ag/PSi-1DPC at n_v_ = 1.1.

In Fig. 3(A), the reflectance of the metal/PSi-1DPC sensor is plotted at a special case of the void refractive index n_v_ = 1.1. Four different metals (Ag, Al, Au, and Pt) are attached to the top of the PSi-1DPC structure, respectively. The thickness of each one is 40 nm.

The refractive index of metals can be described by a complex dielectric function. According to the Drude model, the dielectric function of a metal can be given by the following relation^45,46^,

5 $${{\rm{\epsilon }}}_{{\rm{m}}}=1-\frac{{{\rm{\omega }}}_{{\rm{p}}}^{2}}{{\rm{\omega }}({\rm{\omega }}+{\rm{i}}\,{\rm{\gamma }})}={{\rm{\epsilon }}}_{1}+{\rm{i}}{{\rm{\epsilon }}}_{2}.$$

where *ω*_*p*_ is the plasma frequency and *γ* is the damping frequency term (inverse of the relaxation time). ϵ_1_ and ϵ_2_ are the real and imaginary part of the dielectric function, respectively. Thus, the refractive index of a metal is given by the following relation ${n}_{m}=\sqrt{{{\epsilon }}_{m}}$.

Table 2 summarizes the ω_p_ and γ values for Ag, Au, Al, and Pt, respectively^47^.

**Table 2:** Drude model parameters for metals that used in the calculations^47^.

Metal	Plasma ω_p_ x10^11^ (rad/Sec)	Damping *γ* x10^11^ (rad/Sec)
Al	224107.9	1242.952
Au	137101.3	404.9009
Ag	136913	273.4494
Pt	78155.29	1050.859

It is important to mention that attaching metal to the PSi-1DPC structure will induct the appearance of resonant wavelengths as shown in Fig. 3(A). Those resonant wavelengths or Tamm resonance is due to the interference of electromagnetic waves at the interface between the PSi layers and the metals^48^.

Also, Fig. 3(B) shows a comparison between the reflectance of a PSi-1DPC and Ag/PSi-1DPC at a void refractive index n_v_ = 1.1. It can be seen that the width of the photonic band gap of the Ag/PSi-1DPC is wider compared with the PSi-1DPC due to the existence of the Ag layer on the top of the PSi-1DPC. Also, Fig. 3(B) shows a narrow peak (resonant mode) inside the photonic band gap of the Ag/PSi-1DPC at λ_r_ = 6.7965 µm, which is called Tamm/Fano resonance. Furthermore, the number of ripples in the reflectance spectrum outside the photonic band gap of the PSi-1DPC and Ag/PSi-1DPC is equal.

Moreover, the reflectance of the four metals on the PSi-1DPC at different n_v_ values is shown in Fig. S3 in the supplementary data. As shown in Fig. S3(A) in the supplementary data, with increasing n_v_, the intensity of the resonant wavelengths was decreased and red-shifted towards the higher wavelengths. Also, the photonic band gaps became flat and had a narrower FWHM due to the damping properties of Ag. Moreover, Fig. S3(B) represents the reflection spectrum of the Au/PSi-1DPC. The Au/PSi-1DPC spectrum is similar to the Ag/PSi-1DPC spectrum, this is due to the great convergence in the optical properties between Ag and Au as indicated in Table 2. A motivating phenomenon appeared when the Al layer attached to the PSi-1DPC sensor structure as shown in Figs 3(A) and S3(C,D) in the supplementary data. Not only resonant peaks appeared due to Tamm resonance, but also Fano resonance appeared for each n_v_ value. As is well-known, Fano resonance is asymmetric line shape and results from the interference between slow-varying background (Here is the PSi-1DPC mode) and narrow-band resonance (Tamm resonance)^36,49^. Fano resonance appeared in Al/PSi-1DPC because the damping constant of Al is greater than of Ag or Au. So that the photonic narrow-band (resonance mode) appears clearly in the case of Al than Ag, which in turn produces Fano resonance. Confirming the inspections made above, Fano resonance is sharply observed when Pt is attached to the PSi-1DPC structure as shown in Figs 3(A) and S3(D). The Pt is characterized by high damping constant compared with Al, Au or Ag, which then enhance the appearing of Fano resonance for each n_v_ much clearer. Therefore, Fano resonance in the case of using Pt is clearer than other metals. However, the Pt/PSi-1DPC has a high bandwidth (FWHM) which decrease the values of quality factor and figure of merit, hence, the sensor performance will decrease.

### Sensor analysis

The efficiency and performance of any sensor type are determined by the values of many parameters such as the sensitivity (S), the figure of merits (FOM) and the quality factor (Q). These parameters can be obtained using the following expressions^5,50^.

6 $${\rm{The}}\,{\rm{sensitivity}}\,\,{\rm{S}}={\rm{\Delta }}{\rm{\lambda }}/{\rm{\Delta }}{\rm{n}},$$

7 $${\rm{The}}\,{\rm{figure}}\,{\rm{of}}\,{\rm{merit}}\,\,{\rm{FOM}}={\rm{S}}/{\rm{FWHM}},$$

8 $${\rm{The}}\,{\rm{quality}}\,{\rm{factor}}\,\,{\rm{Q}}={{\rm{\lambda }}}_{{\rm{r}}}/{\rm{FWHM}}.$$

where, the resonance refractive index and wavelength of the air were used as references to calculate Δλ and Δn for the different media, i.e., ${\rm{\Delta }}{\rm{\lambda }}={{\rm{\lambda }}}_{{\rm{r}}}({\rm{void}})-{{\rm{\lambda }}}_{{\rm{r}}}({\rm{air}})$ and ${\rm{\Delta }}{\rm{n}}={{\rm{n}}}_{{\rm{v}}}-{\rm{n}}({\rm{air}})$. The FWHM is the full width at half maximum of the resonant peak.

**Figure 4:**
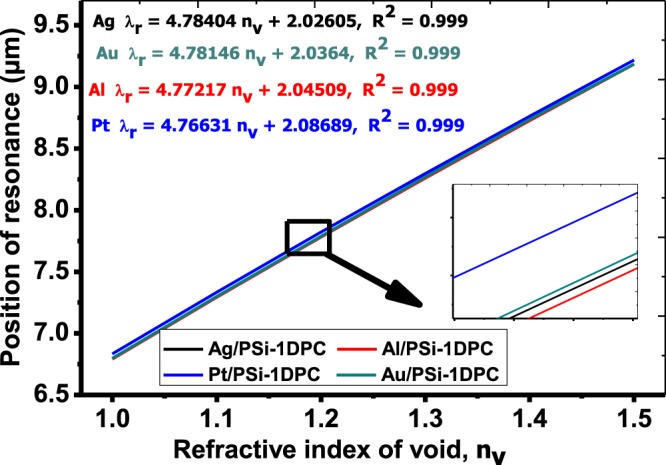
The position of the resonance peak as a function of the void refractive index for Pt/PSi-1DPC, Al/PSi-1DPC, Au/PSi-1DPC and Ag/PSi-1DPC sensor structures.

Figure 4 shows the position of the resonant peak wavelength as a function of n_v_. The positions of the resonant peak are increased linearly with the void refractive index increment for the four metals. The linear fitting of the simulation data for Ag/PSi-1DPC, Au/PSi-1DPC, Al/PSi-1DPC and Pt/PSi-1DPC can be given according to the following equations,

9 $${{\rm{\lambda }}}_{{\rm{r}},{\rm{Ag}}}(\mu {\rm{m}})=4.78404\,{{\rm{n}}}_{{\rm{v}}}({\rm{RIU}})+2.02605\,\,({{\rm{R}}}^{2}=0.999),$$

10 $${{\rm{\lambda }}}_{{\rm{r}},{\rm{Au}}}(\mu {\rm{m}})=4.78146\,{{\rm{n}}}_{{\rm{v}}}\,({\rm{RIU}})+2.0364\,({{\rm{R}}}^{2}=0.999),$$

11 $${{\rm{\lambda }}}_{{\rm{r}},{\rm{Al}}}(\mu {\rm{m}})=4.77217\,{{\rm{n}}}_{{\rm{v}}}\,({\rm{RIU}})+2.04509\,({{\rm{R}}}^{2}=\,0.999),$$

12 $${{\rm{\lambda }}}_{{\rm{r}},{\rm{Pt}}}(\mu {\rm{m}})=4.76631\,{{\rm{n}}}_{{\rm{v}}}\,({\rm{RIU}})+2.08689\,({{\rm{R}}}^{2}=0.999).$$

where the RIU is the refractive index unit. The R^2^ is the square of the correlation coefficient between the linear fitting and the simulation data. The calculated R^2^ is equal to 0.999 for all fitting lines. Hence, high linearity of the suggested biosensor can be obtained. The slope of each fitting line indicates the sensitivity of the Metal/PSi-1DPC. A sensitivity of about 4.76631, 4.77217, 4.78146 and 4.78404 µm/RIU for Pt/PSi-1DPC, Al/PSi-1DPC, Au/PSi-1DPC and Ag/PSi-1DPC, respectively.

The proposed metal/PSi-1DPC structure based on Tamm/Fano resonance allows greater and precise control for the measurements of the refractive index than previously reported sensor structures. These sensors include SPR-based optical fiber sensor described by Cennamo *et al*. (1325 nm/RIU)^51^, Mach-Zehnder interferometer sensor fabricated by Vanita and Vinod (380 nm/RIU)^52^, 1DPC single nanobeam air-mode cavity proposed by Daquan Yang *et al*. (537.8 nm/RIU)^53^, porous silicon microcavity sensor presented experimentally by R. Caroselli *et al*. (1000 nm/RIU)^28^ and 2D-1DPC slab biosensor suggested by El-Beheiry *et al*. (902 nm/RIU)^54^.

In order to design a high-performance biosensor, the resonance peak should be sharp and has high-quality factor. The high-quality factor values lead to accurate sensor measurements and improve the wavelength resolution^55^.

Using Eqs (9–12), the S, FWHM, FOM and Q are calculated at the peak of Tamm/Fano resonance for the metal/PSi-1DPC structure. As shown in Table 3, these parameters were calculated beside the resonance mode (R_r_) for the four metals Ag, Au, Al and Pt at n_v_ = 1.1, respectively. Also, we can see the effects of the void refractive index on the sensor performance parameters in Table S2 in the supplementary data. From this table, the average sensitivity for all metals is about 4.9 µm/RIU (4900 nm/RIU). Not only we obtained high values for the sensitivity, but also for FOM and Q as well. The FOM and Q determine the efficiency and performance of sensors. At n_v_ = 1.1 in the Ag/PSi-1DPC sensor, the Q and FOM have the highest values of about 2149 and 1477.54 RIU^−1^, respectively. The reason of these high values is due to the small broadening in the resonance curve of each Metal/PSi-1DPC structure. Moreover, the trend of all observed calculations is that for higher n_v_ values the PSi-1DPC sensor has the lowest sensitivity for the four metals.

**Table 3:** The values of S, R_r_, FOM, FWHM and Q at n_v_ = 1.1 for the Ag/PSi-1DPC, Au/PSi-1DPC, Al/PSi-1DPC and Pt/PSi-1DPC sensors, respectively.

Metal	λ_r_ (µm)	R_r_ (%)	FWHM (µm)	S (µm/RIU)	FOM (RIU^−1^)	Q
Ag	7.2993	88.43	0.003396	5.018	1477.54	2149.27
Au	7.2997	91.52	0.00496	5.0924	1026.665	1471.65
Al	7.29365	98.74	0.014513	5.0315	346.68	502.55
Pt	7.3348	76.74	0.041341	5.013	121.25	177.42

### Effects of the refractive index and damping constants of metals on the sensor performance

The real and imaginary parts of the refractive index for Ag, Au, Al, and Pt are presented in the supplementary data in Fig. S1. The refractive index is plotted in the wavelength range from 6.35 to 9.85 μm. From the Figs 3(A) and S3, the broadening in the resonance peak in the case of Pt, Al, and Au than Ag is due to their relatively high imaginary part of the dielectric constant (high damping constant). The width of the resonance peak strongly depends on the damping constant of metals^56,57^. Hence, the value of FWHM_Pt/PSi-1DPC_ > FWHM_Al/PSi-1DPC_ > FWHM_Au/PSi-1DPC_ > FWHM_Ag/PSi-1DPC_ at same the refractive index of void as seen in Table 3.

Actually, the performance of a sensor mainly depends on the detection accuracy which is defined as its ability to determine the resonance wavelength and the refractive index of the sensing medium accurately. The detection accuracy is inversely proportional to FWHM of the reflectance dip (resonance peak)^58^. Therefore, the sensor which has a narrow resonant dip will provide a high detection accuracy of the same measurement^59,60^. From our results, the Ag/PSi-1DPC structure has the highest sensitivity and detection accuracy compared with other sensor structures.

To understand the effects of the damping constants of the metal on the sensor performance more, consider a metal has a plasma frequency *ω*_*p*_ = 136913 × 10^11^ (rad/Sec) and the damping frequency changes from *γ* = 18.8326 × 10^12^ to *γ* = 131.8282 × 10^12^ (rad/Sec).

**Figure 5:**
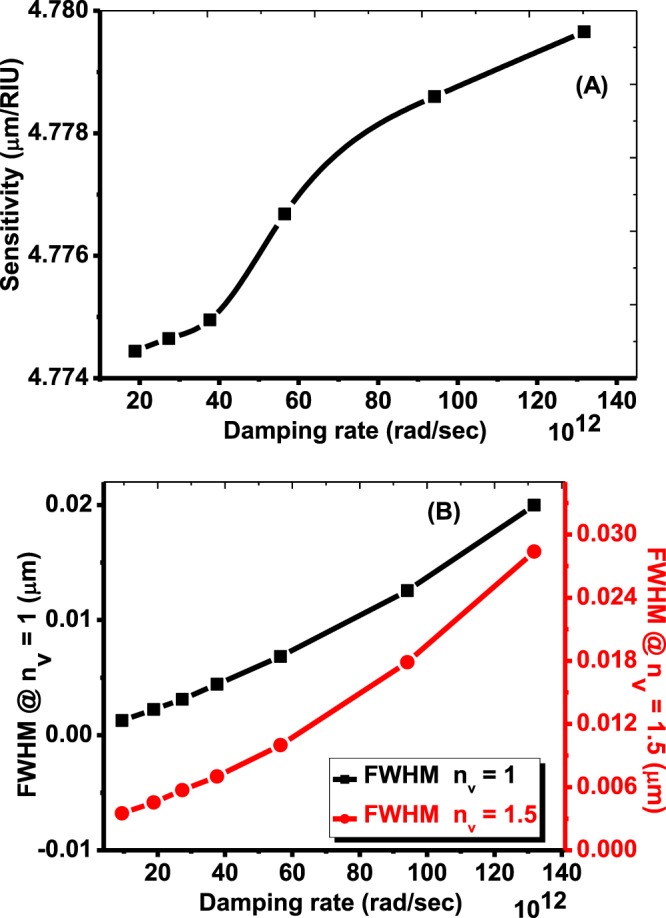
The effects of the damping rate on (**A**) sensitivity (**B**) FWHM.

As seen in Fig. 5(A), the sensitivity increases slightly from 4774.44 to 4779.656 μm/RIU. From Fig. 5(B), the value of FWHM increases from 0.001274 to 0.019988 and from 0.003508 to 0.028379 with increasing the damping constant γ  from 131.8282 × 1012 rad/Sec to the value γ = 18.8326 × 1012 rad/Sec at void refractive indices n_v_ = 1 and n_v_ = 1.5, respectively.

Finally, based on all previous results, the sensitivity is comparable for the four metals due to a few reasons. As well-known, the real part of the dielectric function describes the resonance peak position and the imaginary part describes the broadening of the resonance peak^61^. In our structure, the total thickness of the structure is Si/[PSi_1_/PSi_2_/PSi_3_]^N=25^ is very high (62.5 µm). Also, there is a high change in the effective refractive index for the layers PSi_1_, PSi_2_, and PSi_3_ from 1.6825, 1.381 and 1.220 to 2.0905, 1.8605 and 1.722 with increasing the refractive index of the void from 1 to 1.5, respectively. Hence, the effect of the imaginary part of the metals refractive index on the position of the resonance peak is very small.

## Conclusions

In conclusion, we successfully studied and developed theoretically a high sensitive sensor based on the PSi-1DPC structure. For the first time, the sensing process is based on the displacement of Tamm/Fano resonances by the variation of liquids refractive indices in a metal/PSi-1DPC structure. For Al and Pt, Fano resonance can occurr and couple with Tamm resonance due to the high damping constant of the two elements. Finally, we performed a sensitivity analysis for the proposed PSi-1DPC sensor for the four metals. High sensitivity, quality factor, and figure of merit values were obtained for the four metals Ag, Au, Al, and Pt, respectively. The values of S, Q and FOM for Ag/PSi-1DPC structure are 4784.04 nm/RIU, 2149.27 and 1477.54 RIU^−1^, respectively. Therefore, Fano resonance in the case of using Pt is clearer than other metals. However, the Pt/PSi-1DPC has a high bandwidth (FWHM) which has a negative effect on the values of quality factor and figure of merit which in turn, will decrease the sensor performance. Finally, it would be interesting to realize the proposed devices and to test them for optical sensing.

## Supplementary information

Supplementary Dataset 1
