# Supplementary material for: Ultra-high sensitive 1D porous silicon photonic crystal sensor based on the coupling of Tamm/Fano resonances in the mid-infrared region
Source: Sci Rep. 2019 May 6;9:6973. doi: 10.1038/s41598-019-43440-y (PMC6502859; doi:10.1038/s41598-019-43440-y)
Supplement: Supplementary file 1 — Supplementary Dataset 1 [file 41598_2019_43440_MOESM1_ESM.docx]

**Ultra-high sensitive 1D porous silicon photonic crystal sensor based on the coupling of Tamm/Fano resonances in the mid-infrared region**

Ashour M. Ahmed^1^, Ahmed Mehaney^1^

^1^Physics Department, Faculty of Science, Beni-Suef University, Beni-Suef, 62514, Egypt

*Correspondence should be addressed to A. Mehaney [email: [ahmed011236@scinece.bsu.edu.eg](mailto:ahmed011236@scinece.bsu.edu.eg)]

| Porosity (%)  P1/P2/P3 | Thickness (µm) | Period  N | Ag Metal thickness  (µm) | Sensitivity $S=\Delta\lambda_{r}/0.5$  (nm/RIU) |
| --- | --- | --- | --- | --- |
| P1=0.65  P2=0.77  P3=0.90 | d1=0.220  d2=0.280  d3=0.600 | N=25 | dm=0.050 | 2106 |
| P1=0.65  P2=0.77  P3=0.90 | d1=0.220  d2=0.280  d3=0.700 | N=25 | dm=0.050 | 2318 |
| P1=0.65  P2=0.77  P 3=0.88 | d1=0.220  d2=0.280  d3=0.700 | N=25 | dm=0.050 | 2322 |
| P1=0.65  P2=0.77  P3=0.85 | d1=0.220  d2=0.280  d3=0.700 | N=25 | dm=0.050 | 2322 |
| P1=0.66  P2=0.77  P3=0.88 | d1=0.200  d2=0.300  d3=0.700 | N=25 | dm=0.050 | 2334 |
| P1=0.65  P2=0.77  P3=0.85 | d1=0.300  d2=0.200  d3=0.700 | N=16 | dm=0.050 | 2282 |
| P1=0.65  P2=0.77  P3=0.85 | d1=0.400  d2=0.200  d3=0.700 | N=20 | dm=0.050 | 2418 |
| P1=0.65  P2=0.77  P3=0.85 | d1=0.400  d2=0.200  d3=0.700 | N=25 | dm=0.050 | 2418 |
| P1=0.65  P2=0.77  P3=0.85 | d1=0.400  d2=0.300  d3=0.700 | N=25 | dm=0.050 | 2622 |
| P1=0.65  P2=0.77  P3=0.85 | d1=0.400  d2=0400  d3=0.700 | N=25 | dm=0.010 | 2800 |
| P1=0.65  P2=0.77  P3=0.85 | d1=0.400  d2=0400  d3=0.700 | N=25 | dm=0.050 | 2820 |
| P1=0.65  P2=0.77  P3=0.85 | d1=0.400  d2=0.500  d3=0.700 | N=25 | dm=0.010 | 3002 |
| P1=0.65  P2=0.77  P3=0.85 | d1=0.400  d2=0.500  d3=0.700 | N=25 | dm=0.050 | 3022 |
| P1=0.65  P2=0.77  P3=0.85 | d1=0.400  d2=0.500  d3=0.700 | N=15 | dm=0.050 | 3022 |
| P1=0.65  P2=0.77  P3=0.85 | d1=0.400  d2=0.600  d3=0.700 | N=25 | dm=0.050 | 3220 |
| P1=0.65  P2=0.77  P3=0.85 | d1=0.400  d2=0.650  d3=0.700 | N=25 | dm=0.050 | 3320 |
| P1=0.65  P2=0.77  P3=0.85 | d1=0.400  d2=0.650  d3=0.700 | N=20 | dm=0.050 | 3320 |
| P1=0.65  P2=0.77  P3=0.85 | d1=0.400  d2=0.700  d3=0.700 | N=25 | dm=0.050 | 3418 |
| P1=0.65  P2=0.77  P3=0.85 | d1=0.400  d2=0.800  d3=0.700 | N=25 | dm=0.015 | 3608 |
| P1=0.65  P2=0.77  P3=0.85 | d1=0.400  d2=0.800  d3=0.700 | N=25 | dm=0.050 | 3616 |
| P1=0.65  P2=0.77  P3=0.85 | d1=0.400  d2=0.800  d3=0.800 | N=25 | dm=0.050 | 3818 |
| P1=0.65  P2=0.77  P3=0.85 | d1=0.400  d2=0.800  d3=1.000 | N=25 | dm=0.050 | 4228 |
| P1=0.65  P2=0.77  P3=0.85 | d1=0.400  d2=1.000  d3=1.000 | N=25 | dm=0.050 | 4612 |
| **P1=0.65**  **P2=0.77**  **P3=0.85** | **d1=0.500**  **d2=1.000**  **d3=1.000** | **N=25** | **dm=0.040** | **4775** |

**Table 1S.** The sensitivity of Si / [PSi1 / PSi2 / Psi3]^25^ /Ag at different values of thickness and porosity of each layer and the number of layers to get the best sensor performance. The highest sensitivity is marked in the table with **a bold line** to show the most suitable thickness.

**Figure 1S.** (a) Real and (b) Imaginary parts of refractive index for Ag, Au, Al and Pt as a function of wavelength from 6.35 to 9.85 μm.

**Figure 2S.** The reflectance of the ternary PSi-1DPC with different void refractive indices ($n_{v}$ = 1 to 1.5).

**Figure 3S.** The reflectance spectra at different void refractive indices ($n_{v}$ = 1 to 1.5) for (A) Ag/ PSi-1DPC, (B) Au/ PSi-1DPC, (C) Al/ PSi-1DPC, and (D) Pt/ PSi-1DPC

|  | $n_{v}$ | $\lambda_{r}$  (µm) | R_r_  (% ) | $\mathrm{FWHM}$ (µm) | $S$ (µm/RIU) | FOM  ($\mathrm{RIU}^{-1}$) | $Q$ |
| --- | --- | --- | --- | --- | --- | --- | --- |
| Ag/ PSi-1DPC | 1 | 6.7975 | 86.36 | 0.003216 | 0 | 0 | 2113.36 |
|  | 1.1 | 7.2993 | 88.43 | 0.003396 | 5.018 | 1477.54 | 2149.27 |
|  | 1.2 | 7.7876 | 90.03 | 0.003576 | 4.9505 | 1384.32 | 2177.66 |
|  | 1.3 | 8.2633 | 91.82 | 0.003999 | 4.886 | 1221.76 | 2066.27 |
|  | 1.4 | 8.7291 | 93.51 | 0.004657 | 4.829 | 1036.94 | 1874.41 |
|  | 1.5 | 9.185 | 95.08 | 0.005724 | 4.775 | 834.249 | 1604.72 |
| Au/ PSi-1DPC | 1 | 6.7979 | 90.31 | 0.00484 | 0 | 0 | 1404.30 |
|  | 1.1 | 7.2997 | 91.52 | 0.00496 | 5.0924 | 1026.665 | 1471.65 |
|  | 1.2 | 7.7880 | 92.71 | 0.00524 | 4.9878 | 951.860 | 1486.23 |
|  | 1.3 | 8.2705 | 93.93 | 0.00586 | 4.9335 | 841.244 | 1410.26 |
|  | 1.4 | 8.7374 | 94.81 | 0.00627 | 4.8673 | 775.655 | 1392.39 |
|  | 1.5 | 9.1858 | 95.83 | 0.00742 | 4.7907 | 644.897 | 1236.53 |
| Al/ PSi-1DPC | 1 | 6.7905 | 98.71 | 0.013221 | 0 | 0 | 513.60 |
|  | 1.1 | 7.29365 | 98.74 | 0.014513 | 5.0315 | 346.68 | 502.55 |
|  | 1.2 | 7.7824 | 98.77 | 0.01538 | 4.9595 | 322.46 | 506.00 |
|  | 1.3 | 8.25945 | 98.80 | 0.01676 | 4.8965 | 292.14 | 492.79 |
|  | 1.4 | 8.72635 | 98.82 | 0.018291 | 4.839625 | 264.58 | 477.08 |
|  | 1.5 | 9.1843 | 98.84 | 0.019581 | 4.7876 | 244.49 | 469.03 |
| Pt/ PSi-1DPC | 1 | 6.8335 | 74.25 | 0.040041 | 0 | 0 | 170.66 |
|  | 1.1 | 7.3348 | 76.74 | 0.041341 | 5.013 | 121.25 | 177.42 |
|  | 1.2 | 7.8222 | 78.81 | 0.041626 | 4.9435 | 118.76 | 187.91 |
|  | 1.3 | 8.2975 | 80.59 | 0.04358 | 4.88 | 111.97 | 190.39 |
|  | 1.4 | 8.7624 | 82.16 | 0.045463 | 4.82225 | 106.07 | 192.73 |
|  | 1.5 | 9.2183 | 83.61 | 0.047953 | 4.7696 | 99.464 | 192.23 |

**Table 2S.** The values of S, R_r_, FOM, FWHM and $Q$ for the Ag/ PSi-1DPC, Au/ PSi-1DPC, Al/ PSi-1DPC and Pt/ PSi-1DPC sensors, respectively.
